# Cost-effectiveness analysis of brolucizumab versus aflibercept for the treatment of neovascular age-related macular degeneration (nAMD) in Italy

**DOI:** 10.1186/s12913-022-07972-w

**Published:** 2022-04-29

**Authors:** Nicola Ferrante, Daniela Ritrovato, Rossella Bitonti, Gianluca Furneri

**Affiliations:** 1 Novartis Farma S.p.A, Milan, Italy; 2 EBMA Consulting, Melegnano, MI Italy

**Keywords:** Brolucizumab, Neovascular age-related macular degeneration, nAMD, Cost-effectiveness

## Abstract

**Background:**

Age-related macular degeneration (AMD) is a common and chronic eye condition characterized by the presence of progressive degenerative abnormalities in the central retina (macula). Notably, neovascular, or wet, AMD (nAMD) occurs when new, abnormal blood vessels grow under the macula causing scarring of the macula itself and resulting in a loss of central vision, visual distortion, and an impaired capacity of perceiving colour contrast and intensity. Brolucizumab, a new generation anti-vascular endothelial growth factor (anti-VEGF) monoclonal antibody, was approved by the European Medicines Agency for the treatment of nAMD. The aim of this analysis is to evaluate the cost-effectiveness profile of brolucizumab, compared to the main therapeutic alternative available (aflibercept), for the treatment of nAMD.

**Methods:**

The simulation of costs and outcomes was carried out using a Markov model over a time horizon of 15 years. In base-case, treatment effectiveness inputs for brolucizumab and aflibercept were extracted from the HAWK and HARRIER studies and from a network meta-analysis. The Italian National Healthcare Service (NHS) perspective was considered, therefore only healthcare direct costs (treatment acquisition, administration, adverse events, disease monitoring) were analysed. In the alternative scenarios, the societal perspective and a prolonged time horizon were considered. Model robustness was tested through sensitivity analyses.

**Results:**

In the base-case analysis, brolucizumab was dominant over aflibercept (+ 0.11 years QALY gained and -€15,679 costs). Both one-way deterministic and probabilistic sensitivity analyses confirmed the robustness and reliability of base-case results. The results of the probabilistic sensitivity analysis showed that when the willingness to pay is equal to €50,000 per QALY gained, brolucizumab would be dominant in 84% of simulations and in the remaining simulations brolucizumab would be cost-effective compared to aflibercept. Results of the alternative scenarios and sensitivity analyses confirmed the results of base-case.

**Conclusion:**

The cost-utility analysis shows that brolucizumab is dominant over aflibercept. Treatment with brolucizumab reduces the economic impact of nAMD and determined a slight increase of quality-adjusted survival. This analysis gives a high level of confidence that the treatment with brolucizumab would reduce the burden of intravitreal injections, compared to aflibercept, a relevant therapeutic alternative in Italy.

**Supplementary Information:**

The online version contains supplementary material available at 10.1186/s12913-022-07972-w.

## Background

Age-related macular degeneration (AMD) is a common and chronic eye condition characterized by the presence of progressive, degenerative abnormalities in the central retina (macula) [1]. It usually affects people in their 50s and 60s and can reduce of 20–25% quality of life (QoL), compared to healthy elderly people [2, 3]. In Italy, AMD prevalence is about 2.1% of the general population [4].

The aetiology of AMD is still unknown, but some risk factors, such as nutritional habits, cardiovascular diseases, and genetic markers can increase risk of onset [5]. AMD can be either neovascular (nAMD, also known as exudative or “wet”), or non-neovascular (non-nAMD, also referred to as atrophic or “dry”). Wet AMD is less common (10–15% of all cases) but generally more serious than dry AMD. To date, nAMD is the only form that can be pharmacologically treated [1, 6, 7]. Current licensed treatment options in nAMD are anti-vascular endothelial growth factor (anti-VEGF) agents: ranibizumab (Lucentis®), aflibercept (Eylea®) and pegaptanib (Macugen®). Furthermore, bevacizumab (Avastin®) has been used in Italy as an off-label drug (law 648/96 [8]).

These treatments have improved prognosis of nAMD patients; however, they may suppress disease neovascular AMD activity just temporarily, and they must be administered on a regular basis to secure sustained effect. Therefore, without regular and effective treatment, progression of nAMD persists and disease burden remains [9].

On February 2020, a new-generation anti-VEGF monoclonal antibody, brolucizumab (brand name: Beovu®), was approved by the European Medicines Agency (EMA) for the treatment of nAMD. In pivotal trials HAWK and HARRIER, brolucizumab demonstrated superior anatomic results with greater fluid resolution and similar best-corrected visual acuity compared to aflibercept [10]. These Phase 3 studies demonstrated that brolucizumab is as (at least) effective and safe as aflibercept and it also reduces the burden of regular IVT injections for patients with nAMD (approximately 50% of brolucizumab-treated eyes had stable Best Corrected Visual Acuity -BCVA- during every 12 weeks -Q12- cycles.) [10].

The objective of this analysis is to evaluate cost-effectiveness of brolucizumab, compared to aflibercept, the most used therapeutic alternative in Italy for the treatment of nAMD, adopting the perspective of the Italian National Health Service (NHS).

## Material and methods

### Model design

A cost-effectiveness analysis was performed to compare costs and clinical outcomes associated with brolucizumab (6 mg), administered as IVT injection every 4 weeks (Q4W) for the first three administrations (loading dose), then continued at 8- or 12-week intervals (Q8W/Q12W; maintenance dose) vs aflibercept (2 mg), administered as IVT injection every 4 weeks (Q4W), for the first three administrations (loading dose), then continued at 8-week intervals (Q8W; maintenance dose). The simulation of costs and outcomes was carried out using a Markov model. This approach has already been described in previous cost-effectiveness models on treatments aimed at improving visual acuity [11, 12].

This “one-eye model” simulates patients’ disease status over time, through health states that are defined by the level of Best Corrected Visual Acuity (BCVA), measured by the Early Treatment Diabetic Retinopathy Study (ETDRS) method in one study/affected eye. Transition probabilities between health states are defined by the treatment status of the eye under assessment and time since treatment initiation.

**Fig. 1:**
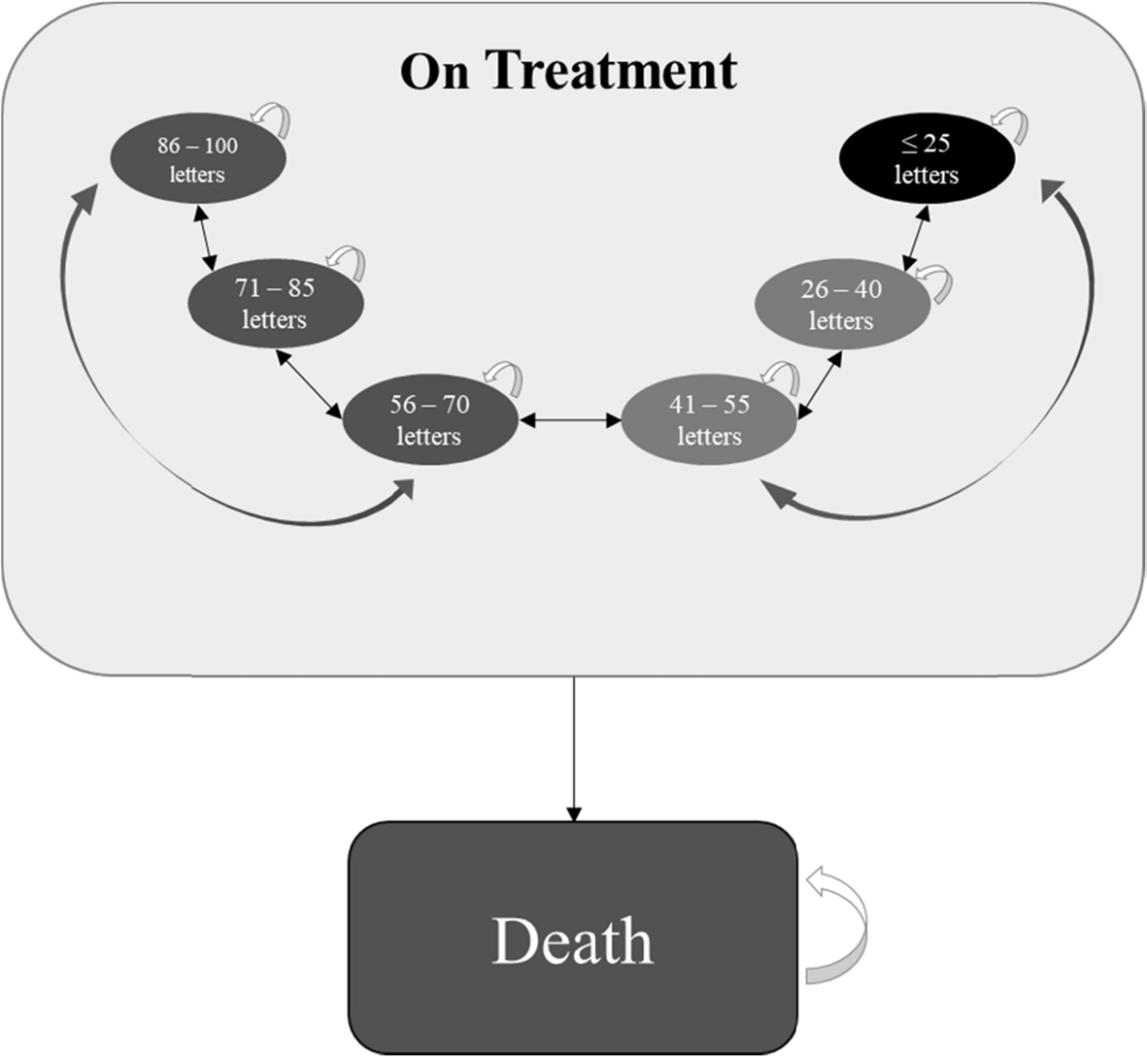
Scheme of the Markov model

Figure 1 illustrates the structure of the model. In each cycle, patients can be in one of the following three macro health states: i) On-treatment: patient on active pharmacological treatment (with either brolucizumab or aflibercept); ii) Off-treatment iii) Death: deceased patient. On-treatment patients can be in one of six health states corresponding to six levels of visual acuity, measured by the ETDRS (Early Treatment Diabetic Retinopathy Study) scale: i) 86–100 letters; ii) 71–85 letters; iii) 56–70 letters; iv) 41–55 letters; v) 26–40 letters; vi) < =25 letters (defined as “blindness”). Patients can move between the various states, or remain in the same state, according to transition probabilities that are treatment-specific and depend on the therapeutic effectiveness of the treatments themselves.

Treatment effectiveness (and permanence of a patient in one of the health states, expressed as BCVA ranges) is measured on the affected and treated eye, which is the eye under evaluation, also called “first eye”. The model estimates the number of patients who will have bilateral disease each year (nAMD in both the study eye and the second eye, also called “fellow eye”). Occurrence of bilateral disease reduces overall patients’ QoL (reduced utilities) and increases treatment costs. However, the health status of the second eye (or fellow eye) does not affect disease progression of the first eye (BCVA in the fellow eye is not considered in this model).

During the “on-treatment phase”, patients receive active treatment, and the evolution of their state of health depends on the effectiveness of the treatment received. In the analysis, it is assumed that treatment efficacy would be different (i.e. better) in Year 1, compared to subsequent years (Year 2+), to reflect clinical outcomes observed in clinical trials and real practice. For Year 2+ the efficacy was based on Year 2 data. Patient discontinuation was not included in the base-case scenario, following clinical expert’s opinion that the rates of discontinuation reported in the clinical trials are significantly higher than those found in clinical practice.

In this model, patients with a mean age of 75.8 years at the beginning of the model are observed over a time horizon of 15 years (equivalent to a lifetime horizon, given age at beginning of simulation). A discount rate of 3.0% was applied to costs and outcomes. The analysis was conducted from the perspective of the National Health Service (NHS) in the base-case analysis. Additional analyses, adopting the Italian societal perspective and using a prolonged time horizon, were run as alternative scenarios.

### Clinical inputs

#### Characteristics of patients at baseline

In this analysis, patients have the same baseline characteristics as in the pooled analysis of studies HAWK and HARRIER [10, 13, 14]. Table 1 summarizes the main characteristics at baseline of the treated population.

**Table 1:** Baseline characteristics [Source: [10, 13, 14]]

Characteristic	Value
Number of patients (n)	1459
Mean age at baseline, years (SD)	75.8 (0.225)
Number of women, N (%)	821 (56.3%)
Number of patients with bilateral disease, N (%)	396 (27.1%)

**Fig. 2:**
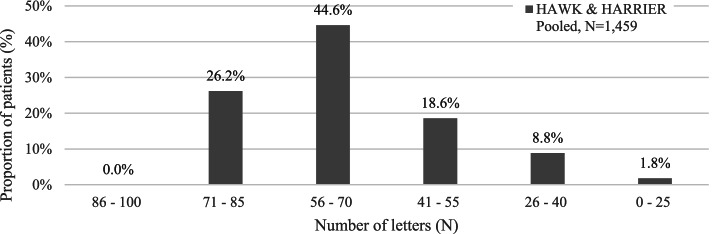
Distribution of patients by baseline BCVA in the study eye [Source: [10, 13, 14]]. *BCVA* Best Corrected Visual Acuity

At the beginning of the simulation (baseline), patients have the same BCVA distribution as in the pivotal clinical studies [10, 13, 14]. Figure 2 shows the distribution of patients by BCVA ranges.

At baseline, in the HAWK & HARRIER studies (pooled analysis), 27.14% of patients had bilateral disease (*N* = 396 out of 1459 patients). This data was included in the model to establish the proportion of patients with bilateral disease at the start of the simulation. The remaining 72.86% of patients with unilateral nAMD would be at risk of developing nAMD in the second eye. In the base-case analysis, a 16.60% annual probability of developing nAMD in the second eye was assumed, as reported in a recent analysis of UK registries [15]. It was assumed that probability remained constant for the duration of the model.

#### Treatment effectiveness

##### BCVA progression in the first two years

In base-case, treatment effectiveness inputs for brolucizumab and aflibercept were extracted from the HAWK and HARRIER studies (pooled analysis [10, 13, 14]) and from a network meta-analysis (NMA) [16]. In these clinical trials, patients were evaluated up to 96 weeks. Change in BCVA from baseline was the key efficacy endpoint [10, 13, 14].

Changes from baseline were expressed in terms of mean change in BCVA ± standard deviation (SD) by treatment arm (independent BCVA variation). A network meta-analysis (NMA) was conducted to estimate the relative treatment effect of every single treatment regimen versus a reference arm aflibercept [16]. NMA estimates for mean change in BCVA for brolucizumab and aflibercept were applied to the baseline mean change in BCVA for aflibercept (Table 2).

**Table 2:** BCVA variation observed in the HAWK and HARRIER studies, regardless of baseline BCVA [Source: [10, 13, 14, 16]]

Treatment	BCVA variation		
	Mean	SD	Number of patients (N)
Brolucizumab (baseline ➔ 52 weeks)	6.50	13.35	730
Aflibercept (baseline ➔ 52 weeks)	7.12	13.51	729
Brolucizumab (52 ➔ 96 weeks)	−0.61	7.18	730
Aflibercept (52 ➔ 96 weeks)	−1.05	8.09	729

*BCVA* Best Corrected Visual Acuity, *SD* Standard deviation

Table 2 shows the efficacy results (BCVA change from baseline) in the brolucizumab and aflibercept arms, regardless of baseline BCVA.

Since complete datapoints to simulate Year 2 were not available, clinical efficacy observed during the first 44 weeks of Year 2 (weeks 53–96) in the HAWK and HARRIER trials [10, 13, 14], intended as BCVA gain or loss (compared to BCVA at week 52), was scaled back to 52 weeks. To adjust 44-week trial data in Year 2 (96 weeks together) to the full year (i.e. 52 weeks), it was assumed that the VA gain/loss would continue progress at the same rate for the remaining 8 weeks.

##### Transition probabilities

The transition matrices between health states of the Markov model were calculated using the BCVA change (observed with brolucizumab and aflibercept in HAWK and HARRIER studies [10, 13, 14]), expressed as the number of letters gained or lost compared to baseline, by transforming these parametric data into transition probabilities.

Transitioning is only possible up to two health states up or down in any given model cycle.

To estimate the probability of gaining or losing a number of letters, the change in BCVA was assumed to follow a normal distribution with the estimated mean and SD, accordingly to the approach proposed by Hodgson et al. 2017 [17]. Using the normal cumulative density function, it is possible to estimate, given the mean and VA letter change cut-offs, the probability of moving across these cut-offs. It was also assumed that BCVA change cut-offs of 7.5 and 22.5 letters would correspond to transitions of one and two health states respectively (Table 3).

**Table 3:** Mapping of the probabilities for the parametric estimation

Probability density estimation	Transition in the model
Gaining ≥22.5 letters	Gaining 2 health states
Gaining 7.5 to 22.5 letters	Gaining 1 health state
Gain/loss < 7.5 letters	No gain/loss
Losing 7.5 to 22.5 letters	Losing 1 health state
Losing ≥22.5 letters	Losing 2 health states

Starting from the distribution of patients by baseline BCVA and calculating the above-mentioned probabilities of gain or loss of letters, it is possible to construct the transition matrices used in the analysis. Supplementary Table 1, in Supplementary materials, shows the transition matrices for brolucizumab and aflibercept.

#### Safety

In the base-case analysis, the incidence of adverse events was calculated separately for brolucizumab 6 mg and aflibercept 2 mg, starting from the pooled data of the HAWK and HARRIER studies at 96 weeks which were adjusted to obtain annual incidences [16]. Only serious ocular events were considered in the analysis. Table 4 lists the observed adverse events and their incidences.

**Table 4:** Incidence of treatment-related adverse events at 96-week pooled from HAWK and HARRIER [Source: [16]]

Adverse event	Brolucizumab 6 mg (***n*** = 730)			Aflibercept 2 mg (***n*** = 729)		
	Number of patients with AEs (N)	Incidence (%)	SE	Number of patients with AEs (N)	Incidence (%)	SE
Cataract	2	0.15%	0.14%	1	0.07%	0.10%
Endophthalmitis	4	0.30%	0.2%	1	0.07%	0.10%
Intraocular inflammation	6	0.45%	0.2%	0	0.00%	0.00%
Detachment of the retina	2	0.15%	0.1%	2	0.15%	0.14%
Retinal pigment epithelial tear	2	0.15%	0.1%	0	0.00%	0.00%
Retinal tear	2	0.15%	0.1%	1	0.07%	0.10%

*AEs* Adverse events, *SE* Standard error

#### Mortality

At any time and health state, patients could move to the death health state.

The progression to the “Death” state was estimated using the mortality rates of the general population, using the Italian mortality tables (year 2018), specific for sex and age (source: ISTAT [18]), and adjusted by the additional risk of any VA-related mortality [19].

### Utility inputs

In the model, each visual acuity state was associated with one utility score (Table 5). These utility scores were extracted from the study conducted by Hodgson et al. 2017 [17].

**Table 5:** Utilities associated with the health states of the model [Source: [17]]

Health state (N of letters)	Utility^a^
86 to 100	0.92
71 to 85	0.82
56 to 70	0.72
41 to 55	0.63
26 to 40	0.53
0 to 25	0.40

^a^the study Hodgson et al. 2017 [17] estimated the relationship between QoL and visual acuity using two regression models: one for the QoL associated with better seeing eye (BSE), one for the QoL associated with worse seeing eye (WSE). In the present analysis, only the second regression model was used, assuming that in clinical trials the drug treatment was always carried out on the eye with worse visual acuity

Disutility related to discomfort from intravitreal injection was applied. This is related to the potential anxiety patients may experience in the days preceding the treatment injection, and discomfort or pain after the treatment. Consistent with NG82 National Institute for Health and Care Excellence [20, 21], 100% utility loss is assumed to last for 1 day for only 50% of all patients. This is equivalent to a loss of ~ 0.0137% of a patient’s regular expected QALY for each injection in a year. Finally, it was assumed that treatment-related adverse events (TRAEs) may reduce patients’ QoL. This effect was quantified through a temporary disutility (the coefficient is subtracted from the utility of the state of health). Table 6 shows the disutility coefficients used in the model. Each AE-related disutility was applied for a certain duration. Among the assumptions, note that there were two adverse events, retinal tear, and intraocular inflammation, which there were assumed to have no impact on QoL (disutility = − 0.00).

**Table 6:** AE-related disutilities included in the model [Source: [22]]

	Description	Disutility	Duration	Source
Additive disutility	Cataract	−0.142	1 month	(Brown et al. 2007) [22]
	Endophthalmitis	−0.3	20% 1 year, 80% 1.5 months	
	Intraocular inflammation	−0.044	1 month	
	Detachment of the retina	−0.27	3 months	
No disutility	Retinal pigment epithelial tear	−0.00	–	No impact
	Retinal tear	−0.00	–	No impact

### Cost inputs

In the base-case (NHS perspective), the following costs were included in the analysis: i) costs of treatment acquisition; ii) costs of treatment administration; iii) costs of disease monitoring; iv) direct costs associated with vision loss; v) costs of treatment-related adverse event (TRAE) management.

Table 7 lists all cost input data and resource consumption assumptions used in the model.

**Table 7:** Cost input included in the analysis

Type	Description	Value	Source
Treatment acquisition	Acquisition cost – Brolucizumab (€/vial)^a^	€680.00^a^	[23, 24]
	Acquisition cost – Aflibercept (€/vial)^a^	€740.00^a^	
	Number of injections, Year 1 – Brolucizumab (N/year)	6.66	[10, 13, 14]
	Number of injections, Year 1 – Aflibercept (N/year)	7.23	
	Number of injections, Year 2 – Brolucizumab (N/year)	4.84	
	Number of injections, Year 2 – Aflibercept (N/year)	5.58	
	Number of injections, Subsequent years – Brolucizumab (N/year)	4.84	
	Number of injections, Subsequent years – Aflibercept (N/year)	5.58	
Treatment administration	Administration cost of anti-VEGF drug (€/administration)	€247.20	Elaboration from [25]
Disease monitoring	Unit cost - Optical Coherence Tomography (€)	€36.07	Elaboration from [25]
	Resource use - Optical Coherence Tomography (N/anno)	1.00	
TREA management	Unit cost (€) - Cataract	€994.00	DH 039 [27]
	Unit cost (€) - Endophthalmitis	€1522.00	DH 042 [27]
	Unit cost (€) - Intraocular inflammation	€20.66	Tariff Code 89.7 [28]
	Unit cost (€) - Detachment of the retina	€1491.00	DH 036 [27]
	Unit cost (€) - Retinal pigment epithelial tear	€1491.00	
	Unit cost (€) - Retinal tear	€1491.00	
Vision loss	Total costs direct of blindness (€)	€7856	[26]
	Total costs direct of low vision (€)	€1964	
	Total costs (direct and indirect) of blindness (€)	€17,897	
	Total costs (direct and indirect) of low vision (€)	€4474	

*DH* Day hospital, *TRAE* Treatment-related adverse event, *VEGF* Vascular endothelial growth factor

^a^The analysis was conducted using the ex-factory price including the mandatory legal discounts applied to public structures of the Italian NHS

To calculate the costs of treatment acquisition, the unit cost of a vial (ex-factory price per vial [23, 24], including the mandatory legal discounts applied to public structures of the Italian NHS) was multiplied by the number of vials per treatment [10, 13, 14] (cost per year) (Table 7).

Costs of treatment administration were added to treatment acquisition costs to estimate the overall cost of drug therapy. In the analysis, administration costs did not depend on the type of anti-VEGF drug administered. The unit cost of the intravitreal administration was calculated as the weighted average of the regional outpatient service fees using PAC (Complex Outpatient Package) with code 14.75 “Intravitreal injection of therapeutic substances; including: specialist visit, pre-operative examinations, enrolment, intervention, check-up visit; excluding cost of the drug” [25].

Disease monitoring requires the patient to undergo specialist control visits, including optical coherence tomography (OCT). It was assumed that these visits would be conducted once a year. Costs of disease monitoring were calculated as the weighted average of the regional outpatient service fee “Optical Coherence Tomography”, in the regions where the service is currently reimbursed (Abruzzo, Calabria, Campania, Lazio, Molise, Puglia and Sardinia) [25].

The costs of TRAE management were calculated by multiplying the unit costs of adverse events in Italian practice [16], by the incidence rates reported in Table 7. It was assumed that adverse events can be managed by a specialist doctor, or in a day-hospital setting, depending on the type of event.

In the alternative scenario (societal perspective), the indirect costs associated with the vision loss (blindness and low vision) were included. The article published in 2011 by Muscio et al. [26] on direct and indirect costs attributable to blindness in Italy was taken as reference to estimate the costs of blindness (Table 7). Since no reliable sources are available for the costs of low vision, it was arbitrarily assumed that these amount to 25% of the costs of blindness.

### Sensitivity analysis

Deterministic (one-way) and probabilistic sensitivity analyses were carried out to identify the input values with the largest effect on incremental cost-effectiveness ratio (ICER).

For the deterministic sensitivity analysis, the baseline value of each parameter was modified to the upper and lower limits of its 95% confidence interval (95% CI) or using the standard error (SE). In the absence of available data, a variation of ±10% from the baseline value was used. It was decided to also vary the economic data by ±10% (e.g. TRAE management costs), although the latter were not (plausibly) affected by a high level of uncertainty.

Finally, a probabilistic sensitivity analysis was performed, simultaneously and randomly varying the values of all model parameters (1000 replications). For the probabilistic analysis, the following probability distributions were used: beta for probabilities, proportions, incidences, and rates; gamma for costs; normal for utilities.

## Results

### Base-case analysis

Results of the base-case analysis are shown in Table 8. Overall, treatment with brolucizumab slightly increased quality adjusted survival (+ 0.11 years QALY gained), compared to aflibercept. On parallel, treatment with brolucizumab resulted in lower direct healthcare costs (−€15,679), compared to aflibercept.

**Table 8:** Results of cost-effectiveness analysis: base-case

	Brolucizumab (A)	Aflibercept (B)	Difference (A-B)
Outcomes			
Life years (LYs)	9.00	9.00	0.00
Quality adjusted life years (QALYs)	6.43	6.32	0.11
Direct costs (€)			
Treatment acquisition costs (€)	46,821	58,265	- 11,444
Treatment administration costs (€)	15,130	17,277	- 2147
Disease monitoring costs (€)	1663	1895	- 232
TREA management costs (€)	115	47	68
Direct costs of blindness (€)	7632	8157	- 525
Direct costs of low vision (€)	4626	6025	- 1399
Total direct costs (€)	75,988	91,666	−15,679
Incremental cost-effectiveness ratio (ICER)			
ICER (brolucizumab vs aflibercept) (€/QALY)	Brolucizumab dominant		

*ICER* Incremental cost-effectiveness ratio, *LYs* life years, *QALYs* Quality adjusted life years, *TRAE* Treatment-related adverse event

Therefore, brolucizumab was dominant over aflibercept.

### Sensitivity analysis

**Fig. 3:**
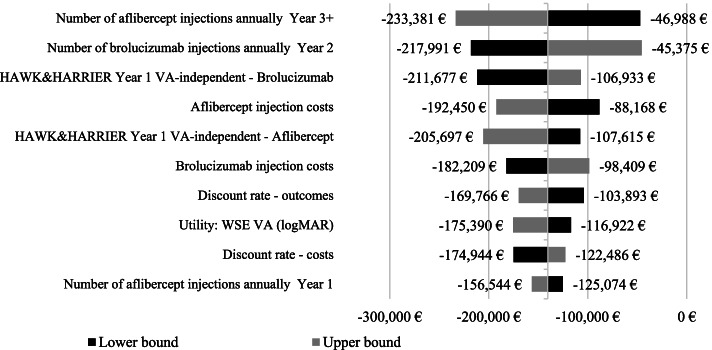
Results of one-way deterministic sensitivity analysis. *VA* Visual Acuity, *WSE* worse seeing eye

Both one-way deterministic and probabilistic sensitivity analyses confirmed the robustness and reliability of base-case results. The results of one-way deterministic analysis are summarized in Fig. 3, that illustrates the 10 parameters / scenarios with the greatest effect on the ICER (base-case ICER: -€140,309 / QALY). The ICER variability was modest (minimum ICER: -€211,677/ QALY gained; maximum ICER: -€88,168/ QALY gained). Brolucizumab remained dominant over aflibercept in all tested scenarios.

**Fig. 4:**
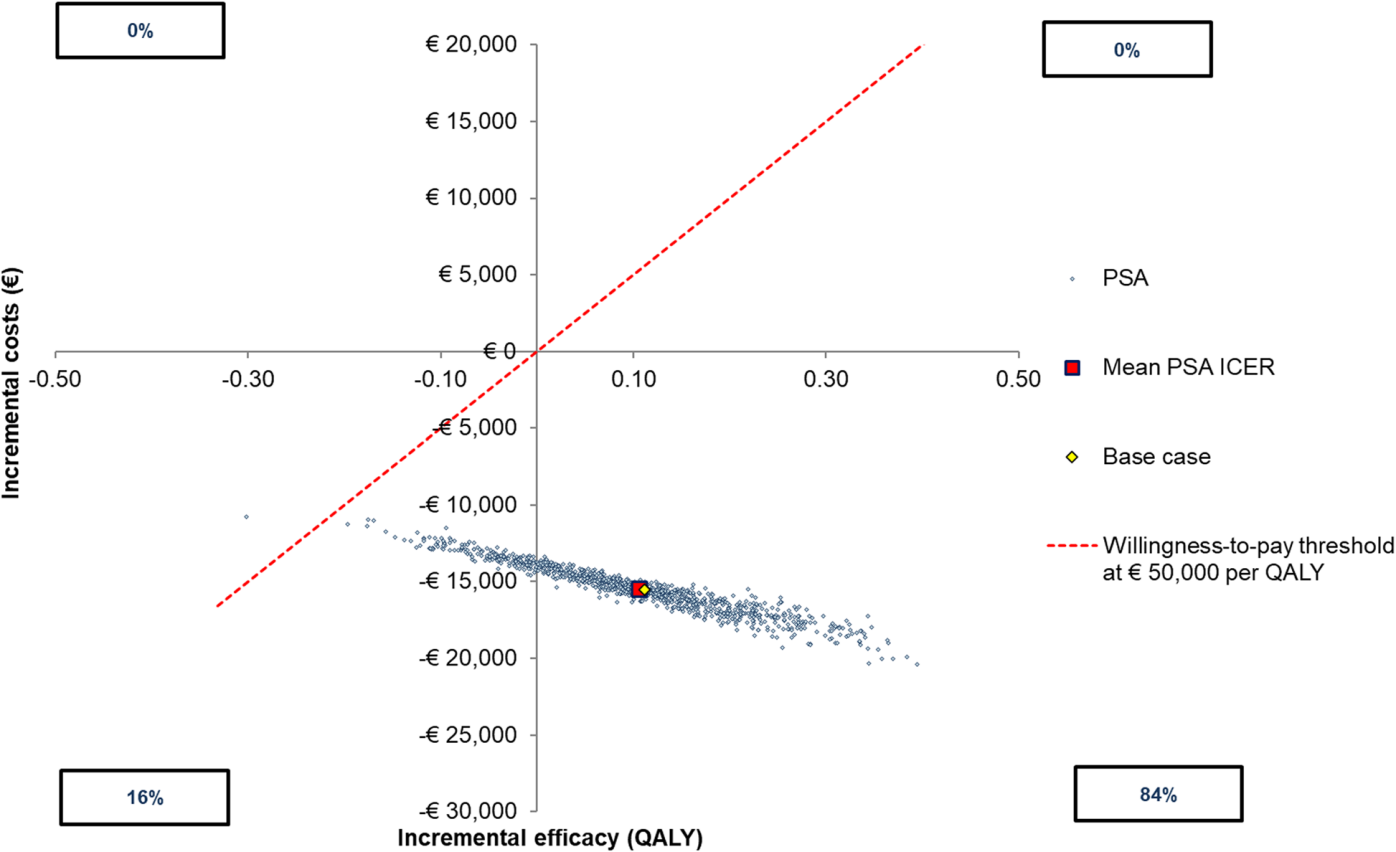
Results of probabilistic sensitivity analysis. *ICER* incremental cost-effectiveness ratio, *QALY* quality-adjusted life-year, *PSA* probabilistic sensitivity analysis

The results of the probabilistic sensitivity analysis are shown in Fig. 4 (scatter plot). The scatterplot (Fig. 4) showed that when the willingness to pay (WTP) is equal to €50,000 per QALY gained, brolucizumab would be dominant in 84% of simulations and in the remaining simulations (16%) brolucizumab would be cost-effective compared to aflibercept.

### Alternative scenarios

In the alternative scenario, brolucizumab was compared vs aflibercept adopting the societal perspective; results are shown in Table 9 Results of the alternative scenario were better than the base-case as, including social costs in the analysis, a slight increase in savings was generated (−€18,137 vs. -€15,679).

**Table 9:** Results of the alternative scenario analysis

	Brolucizumab (A)	Aflibercept (B)	Difference (A-B)
Outcomes			
Life years (LYs)	9.00	9.00	0.00
Quality adjusted life years (QALYs)	6.43	6.32	0.11
Direct and indirect costs (€)			
Treatment acquisition costs (€)	46,821	58,265	- 11,444
Treatment administration costs (€)	15,130	17,277	- 2147
Disease monitoring costs (€)	1663	1895	- 232
TREA management costs (€)	115	47	68
Costs of blindness (€)	17,386	18,582	- 1196
Costs of low vision (€)	10,539	13,725	- 3186
Total costs (€)	91,654	109,791	−18,137
Incremental cost-effectiveness ratio (ICER)			
ICER (brolucizumab vs aflibercept) (€/QALY)	Brolucizumab dominant		

*ICER* Incremental cost-effectiveness ratio, *LYs* Life years, *QALYs* Quality adjusted life years, *TRAE* Treatment-related adverse event

In another alternative scenario, where the analysis was conducted prolonging the time horizon from 15 to 40 years, brolucizumab remained dominant vs aflibercept.

## Discussion

This cost-utility analysis shows that brolucizumab is dominant over aflibercept if the net prices applied to the Italian NHS (including estimated mandatory legal discounts) are considered. Treatment with brolucizumab generated savings on acquisition costs (− € 11,444 per patient) and costs associated with vision loss (− € 1924 per patient), and also determined a slight increase of quality-adjusted survival (+ 0.11 QALY).

As for all economic evaluations, assessment of methodology and assumptions is crucial to pressure-test the validity of findings. In our view, few aspects of this analysis should be investigated: i) variability in frequency of administrations; ii) treatment effectiveness after Year 2; iii) quantification of costs associated with vision loss; iv) assumptions on discontinuation; v) “one-eye model” design.

Regarding the first point (utilization rates), evidence from the Italian National Observatory on the Use of Medicines (OsMed) shows that annual number of anti-nAMD injections in real world (3.6 injections/year) is lower than utilization observed in brolucizumab pivotal trials [29]. This lower treatment rate is due to several factors: i) limited hospital budget and capacity (there are not sufficient resources and capacity to carry out a complete course of treatment); ii) patient preference (patients may tend to “reject” frequent intravitreal injections); iii) medical considerations (many ophthalmologists believe that a limited number of anti-VEGF injections is effective enough to achieve stable BCVA). For brolucizumab, the recommended posology is every 4 weeks for the first three doses; thereafter, physicians may individualise treatment intervals based on disease activity assessment. To date, real-world data on brolucizumab are limited, and hypotheses on utilization are difficult to make; therefore, an update of this analysis would be needed as soon as real-world evidence will be available.

Regarding the second point (long-term efficacy), a constant effect of brolucizumab was assumed after Year 2. Indeed, the pooled analysis from HAWK & HARRIER studies [10, 13, 14] informs on efficacy up to 96 weeks. There is a chance this assumption would not necessarily reflect reality, and a waning effect would be observed over time. Again, long-term evaluation of brolucizumab in real practice would help addressing this uncertainty in the future.

Regarding the third point (cost of low vision), since no reliable sources were available, assumptions from Muscio et al. study were used and costs of low vision were assumed to be a proportion (25%) of the costs of blindness [26]. Even in this case, an update of this analysis would be worth, as soon as more reliable information about such costs will be available. However, the uncertainty related to this parameter was assessed through deterministic and probabilistic sensitivity analyses. As a matter of fact, different assumptions on costs of low vision did not impact ICER much, and brolucizumab remained cost-saving even in the extreme case of these costs being excluded from the analysis.

To the fourth point (discontinuation), it was assumed that patients did not stop treatment (i.e., no off-treatment patients) with brolucizumab and aflibercept. This assumption was validated with clinical experts; if discontinuation was included in the analysis, then brolucizumab would be cost-effective vs aflibercept, due to higher cost for lower discontinuation rates, but more prolonged efficacy.

Finally, the “one-eye model” design (fifth point) might be seen as a limitation, as it would not adequately capture costs and consequences of patients with bilateral nAMD at baseline, or patients developing nAMD in the second eye later. Also costs and effects of treatment would be different if patients with bilateral nAMD would receive treatment in their better-seeing eye or their worse-seeing eye [11].

In this paper, brolucizumab was compared vs aflibercept, whereas other treatment options are available in the Italian market. The reason to exclude other comparators from this analysis (ranibizumab in particular) is that we aimed to prioritize the only comparison supported by head-to-head evidence. Frequently, cost-effectiveness analyses are criticised because of the uncertainty deriving from indirect treatment comparisons. In this case, there was an opportunity to extract data from a direct comparison (studies HAWK & HARRIER); this is of great value in conditions like nAMD, where there is a need to reduce model uncertainty, which is quite high for the reasons that were mentioned above. However, we believe this is not a major issue, brolucizumab vial cost is lower than ranibizumab vail cost, in Italy; therefore, if we had used the current cost-effectiveness model to compare brolucizumab vs ranibizumab, brolucizumab would have been dominant vs ranibizumab.

## Conclusions

The results of the cost-effectiveness analysis confirm that brolucizumab is a cost-effective option for the treatment of patients affected by neovascular age-related macular degeneration, compared with aflibercept, when evaluated from the Italian NHS perspective. This analysis gives a high level of confidence that treatment with brolucizumab would reduce the burden of intravitreal injections, compared to aflibercept, a relevant therapeutic alternative in Italy. This would translate in reduced budget impact for the Italian NHS, increased preference for patients, who aim to minimize discomfort from intravitreal injection, increased efficiency for hospitals, that need to reduce the per-patient workload to increase capacity and shorten waiting lists. These advantages would make brolucizumab a valuable asset to improve nAMD management in the upcoming years.

## Supplementary Information

**Additional file 1.**

## Data Availability

All data generated or analysed during this study are included in the following published article: Dugel PU, Koh A, Ogura Y, Jaffe GJ, Schmidt-Erfurth U, Brown DM, et al. HAWK and HARRIER: Phase 3, Multicenter, Randomized, Double-Masked Trials of Brolucizumab for Neovascular Age-Related Macular Degeneration. Ophthalmology. 2019; doi:10.1016/j.ophtha.2019.04.017.

## Abbreviations

AEs: Adverse events
AMD: Age-related macular degeneration
anti-VEGF: Anti-vascular endothelial growth factor
BCVA: Best Corrected Visual Acuity
BSE: Better seeing eye
CI: Confidence interval
dB: Decibel
DH: Day hospital
EMA: European Medicines Agency
ETDRS: Early Treatment Diabetic Retinopathy Study
H&H: HAWK and HARRIER
IOP: Intraocular pressure
ICER: Incremental cost-effectiveness ratio
IVT: Intravitreal
ISTAT: Italian Institute of Statistics
LYs: life years
MD: Mean deviation
nAMD: Neovascular age-related macular degeneration
non-nAMD: Non-neovascular age-related macular degeneration
NHS: National Health Service
NICE: National Institute of Health Care and Excellence
NMA: Network meta-analysis
OCT: Optical coherence tomography
OsMed: National observatory on the use of medicines
PAC: Complex outpatient package
Q4W: Every 4 weeks
QALY: Quality-adjusted life years
QoL: Quality of life
RCT: Randomized clinical trial
SD: Standard deviation
SE: Standard error
TRAE: Treatment-related adverse event
UK: United Kingdom
US: United States
VA: Visual acuity
VF: Visual field
WSE: Worse seeing eye
WTP: Willingness to pay
