# Supplementary material for: Cost-effectiveness analysis of brolucizumab versus aflibercept for the treatment of neovascular age-related macular degeneration (nAMD) in Italy
Source: BMC Health Serv Res. 2022 Apr 29;22:573. doi: 10.1186/s12913-022-07972-w (PMC9052557; doi:10.1186/s12913-022-07972-w)
Supplement: Supplementary file 1 — Additional file 1. [file 12913_2022_7972_MOESM1_ESM.docx]

# Supplementary materials

**Supplementary table 1 Transition matrices of the Markov model (brolucizumab and aflibercept) [Source:** [12,15,16]**]**.

| **Brolucizumab** | **from \ to:** | **86 - 100 letters** | **71 - 85 letters** | **56 - 70 letters** | **41 - 55 letters** | **26 - 40 letters** | **0 - 25**  **letters** |
| --- | --- | --- | --- | --- | --- | --- | --- |
| **Year 1** | **86 - 100 letters** | 86% | 13% | 1% | 0% | 0% | 0% |
|  | **71 - 85 letters** | 47% | 38% | 13% | 1% | 0% | 0% |
|  | **56 - 70 letters** | 12% | 35% | 38% | 13% | 1% | 0% |
|  | **41 - 55 letters** | 0% | 16% | 41% | 29% | 13% | 1% |
|  | **26 - 40 letters** | 0% | 0% | 21% | 45% | 27% | 7% |
|  | **0 - 25 letters** | 0% | 0% | 0% | 21% | 45% | 34% |
| **Year 2** | **86 - 100 letters** | 83% | 17% | 0% | 0% | 0% | 0% |
|  | **71 - 85 letters** | 13% | 70% | 17% | 0% | 0% | 0% |
|  | **56 - 70 letters** | 0% | 13% | 70% | 17% | 0% | 0% |
|  | **41 - 55 letters** | 0% | 0% | 13% | 70% | 17% | 0% |
|  | **26 - 40 letters** | 0% | 0% | 0% | 13% | 70% | 17% |
|  | **0 - 25 letters** | 0% | 0% | 0% | 0% | 13% | 87% |

| **Aflibercept** | **from \ to:** | **86 - 100 letters** | **71 - 85 letters** | **56 - 70 letters** | **41 - 55 letters** | **26 - 40 letters** | **0 - 25**  **letters** |
| --- | --- | --- | --- | --- | --- | --- | --- |
| **Year 1** | **86 - 100 letters** | 87% | 12% | 1% | 0% | 0% | 0% |
|  | **71 - 85 letters** | 49% | 37% | 12% | 1% | 0% | 0% |
|  | **56 - 70 letters** | 13% | 36% | 37% | 13% | 1% | 0% |
|  | **41 - 55 letters** | 0% | 18% | 41% | 28% | 12% | 1% |
|  | **26 - 40 letters** | 0% | 0% | 22% | 45% | 26% | 7% |
|  | **0 - 25 letters** | 0% | 0% | 0% | 22% | 45% | 33% |
| **Year 2** | **86 - 100 letters** | 78% | 22% | 0% | 0% | 0% | 0% |
|  | **71 - 85 letters** | 14% | 64% | 22% | 0% | 0% | 0% |
|  | **56 - 70 letters** | 0% | 14% | 64% | 22% | 0% | 0% |
|  | **41 - 55 letters** | 0% | 0% | 14% | 64% | 22% | 0% |
|  | **26 - 40 letters** | 0% | 0% | 0% | 14% | 64% | 22% |
|  | **0 - 25 letters** | 0% | 0% | 0% | 0% | 14% | 86% |
